# Metal Ion Accumulation on Denture Teeth Following Exposure to Chlorhexidine and Different Drinks: A Spectrometric Analysis

**DOI:** 10.3390/ma13092021

**Published:** 2020-04-26

**Authors:** Gerard A. Fischer, Georgios E. Romanos

**Affiliations:** Laboratory for Periodontal-, Implant-, Phototherapy (LA-PIP), School of Dental Medicine, Department of Periodontology, Stony Brook University, Stony Brook, NY 11794, USA

**Keywords:** abrasion, artificial teeth, metal hypersensitivity, nutrition, spectrometry

## Abstract

Denture teeth are used for removable prostheses and implant-supportive restorations. The purpose of this study was to analyze the surface of artificial teeth following exposure to different liquids. Mechanical wear and the cleaning of artificial teeth were also investigated. Two groups of resin teeth were used; original surface (Group A) and surface abrasion/wear (Group B). The teeth were exposed for 24 h to water (control), cola (Group 1), coffee (Group 2), tea (Group 3), chlorhexidine (Group 4), and red wine (Group 5). Baseline measurements of liquids were taken. An x-ray fluorescence spectrometric analysis was performed. Data were evaluated using semi-quantitative descriptive analysis. The data showed approximate increases of peak intensity for Group A1-2-fold Fe, 2-fold Ni, 2.2-fold Zn; for Groups A2 and A3- less than 1.5-fold Fe, Ni, and Zn. Group B yielded similar results; however, the increases in Fe, Ni, and Zn were significantly higher in Groups 1 and 2 (ranged 2-4-fold increases in intensity). Group B3 showed little increase in Fe, Ni, and Zn. Groups A4 and A5 showed intensity increases for Zn. Groups A1–A5 showed reductions in intensity following 30 s of cleaning. A pronounced accumulation of iron, nickel, and zinc was found after exposure to liquids, especially when artificial teeth were worn down. Peak intensities were reduced following 30 s of brushing.

## 1. Introduction

Artificial resin teeth for full or partial removable restorations have become common, especially in older populations, as many aging adults are missing one or more permanent teeth [1]. Artificial teeth in conjunction with both fixed and removable implant-supportive appliances are a popular solution to restore aesthetics and function. The importance of proper care for these teeth through cleaning and dietary habits is well documented, but many patients still neglect their oral healthcare following treatment with these restorations [2]. 

Diet is known to play a crucial role in the health of natural teeth, artificial teeth, and the supporting hard and soft tissues. Previous research has shown that adults who routinely make poor dietary choices are at a greater risk for periodontal disease [3]. Staining can also be an issue when considering the esthetics of anterior teeth. Coffee and red wine are both popular beverages in many countries [4,5]. Frequent consumption of coffee, red wine, cola, and tea is associated with the staining of teeth [6,7,8]. Previous studies have shown that patients who consume these beverages may find it more difficult to restore their natural tooth color through common whitening methods [3]. Some studies have shown that consumption of these beverages may also lead to metal ion accumulation on the surfaces of artificial teeth. Depending on which metals accumulate following beverage consumption, it is possible that these changes to the artificial tooth surface could impact plaque accumulation and cause further biological complications in oral tissues via inflammatory processes [9].

The aim of this study was to analyze the surface of artificial resin teeth following exposure to chlorhexidine and popular beverages such as cola, coffee, tea, and red wine. Mechanical wear and proper cleaning of the artificial teeth were both investigated. Many dental patients experience wear via mechanical, chemical, and parafunctional means. This is significant for both natural and artificial teeth [10]. With this in mind, we exposed both new and worn-down artificial teeth to the previously mentioned liquids. Many patients also neglect proper care for their artificial teeth, so we investigated the effects of brushing these teeth following exposure to liquids [2]. It was hypothesized that exposing artificial resin teeth to various liquids would lead to an accumulation of metal ions, such as nickel. An x-ray fluorescence spectrometer was used to analyze artificial teeth surfaces before and after exposure to liquids.

## 2. Materials and Methods 

### 2.1. X-ray Fluorescence Spectrometry

The experiments of this study were carried out using an X-ray fluorescence spectrometer made by Rigaku (Tokyo, Japan). The specific model we utilized was a NEX DE Energy Dispersive X-ray Fluorescence Spectrometer (Rigaku, Austin, TX, USA). This model utilizes the Windows®-based QuantEZ software for data processing, a 60 kV X-ray tube as the source, and a FAST SDD® detector (Amptek, Bedford, MA, USA) for data collection. This technology allowed us to perform a fundamental parameters (FP) analysis of artificial teeth at the elemental level under various conditions. FP analysis is semi-quantitative as it is a method which does not require a standard. It is for this reason that data presented in this study are shown as changes in peak intensities of specific elements rather than specific concentrations of elements. In order to analyze a sample at the elemental level, the spectrometer emits an incidence x-ray which leads to the ejection of an inner shell electron within the atoms of the sample. An outer electron then fills the vacancy, and the spectrometer measures the energy difference, which is specific for each element. The spectrometer calibration was kept up to date following the manufacturer guidelines. Prior to each experiment, the multi-channel analyzer (MCA) and library calibrations were run using the calibration sample included with the machine. The experimental set-up parameters were as follows: a helium atmosphere with a flow rate of 200.00 mL/min which was achieved by keeping the pressure of the tank between 5–7 psi, DE-10 mm, a measuring time of 60 s per atomic number level (high-z, mid-z, low-z), selected FP analysis template based on sample to be tested (either liquid or polymer/resin), default selection of elements to be tested according to selected template, selected counts per second (cps) for units of intensity, power of 12 W, and 60 kV X-ray tube.

### 2.2. Exposure of Denture Teeth to Various Liquids and Spectrometric Analysis

Massad^®^ artificial resin posterior molars made with Polymethyl-methacrylate (PMMA) polymers by Nobilium (Albany, NY, USA) were used throughout this study. Initially, a new artificial tooth was placed in the sample chamber of the spectrometer and analyzed at the elemental level to get a baseline assessment. The tooth was placed such that the camera was aligned with the middle of the occlusal table for each reading throughout the study. Following this baseline analysis, the tooth was exposed to 50mL of one of the liquids analyzed in this study for 24 h consecutively. The exposure was characterized by complete submersion of the tooth in the liquid in order to ensure that the occlusal table, which was the area facing the spectrometer camera for each trial, was in constant contact with the liquid for the full 24 h. The liquids of interest were coffee, cola, black tea (Earl Grey), chlorhexidine, and red wine (Cabernet Sauvignon). Each of these liquids was also placed in the sample chamber in order to get the baseline elemental analyses. This was completed by pouring out a small aliquot of liquid into one of the sample cups followed by the placement of the cup in the chamber for analysis. A new sample cup was used for each liquid to prevent contamination. For each experiment, five artificial resin teeth were exposed to each liquid to gather data from multiple trials. The spectra shown in the results are each taken from one trial and they each display the results of an artificial tooth before and after exposure to a liquid plotted on the same graph. A semi-quantitative analysis of the spectra was performed. A control experiment was carried out to check for variability between different runs of the spectrometer. For this run, a new tooth was analyzed prior to exposure to tap water for 24 h. The tooth was then analyzed, and the results were plotted on the same graph.

### 2.3. Simulation of Abrasion of Denture Teeth

In order to investigate the possible role of surface abrasion in this experiment, new artificial teeth were mechanically worn-down using a conventional high-speed dental handpiece made by Bien Air Dental (Irvine, CA, USA) and an FG super coarse round end tapered diamond drill made by Brasseler USA Dental (Savannah, GA, USA). The drilling was undertaken for 30 s, making sure to strip away the most superficial layer of the occlusal table. Copious water irrigation was used with the handpiece to ensure that the tooth was not burned. The worn-down teeth were then rinsed, dried, and subjected to the same procedure outlined above for liquid exposure. The results of the experiments performed using both the new, and the worn-down teeth were plotted on the same graph. A semi-quantitative analysis of the spectra was then carried out.

### 2.4. Cleaning of Artificial Teeth

In addition to the possible role of wear, we investigated the potential role of tooth brushing on artificial teeth following 24 h of exposure to a liquid. The artificial teeth were analyzed prior to any exposure as well as after 24 h of exposure. The teeth were then immediately cleaned with a conventional soft toothbrush under running tap water for 30 s, and then analyzed once more in the spectrometer. The three spectra were then plotted together for semi-quantitative analysis.

## 3. Results

The spectrometric analysis showed a 1-2-fold increase in peak intensities related to the concentrations of iron, nickel, and zinc when artificial teeth with their original surfaces were exposed to coffee and cola. The peak intensities increased by 2-4-fold for artificial teeth with worn down surfaces following exposure to coffee and cola. The change in peak intensities was not as prominent for either surface following exposure to tea. Exposure of original artificial teeth surfaces to chlorhexidine or red wine yielded an increase in the peak intensity of zinc. The elevated peak intensities for artificial teeth with their original surfaces following exposure to all the liquids in this study were reduced following 30 s of brushing under tap water.

The figures shown in this section are spectra taken from one trial of each experimental group. Data from multiple trials were collected and are summarized in Table 1 and Table 2. The average changes observed in peak intensities for iron, nickel and zinc for each experiment are recorded. The average fold changes in area under the peaks for these elements compared to the change in area under the peak corresponding to Cu are recorded in Table 1 as well. These changes are reflected in the spectra that have been included in this paper. Each spectrum has been scaled to range from 6.0–9.0 KeV of energy shown on the x-axis, and 0–15 counts per second (cps) for the intensity displayed on the y-axis. The elements labeled in these results are iron, nickel, and zinc. While peaks may be notable for copper at approximately 8.0 KeV, they are not labeled, as no distinct patterns or changes were noted throughout the experiments. Because this parameter did not change significantly throughout the experiments, the change in area under the peak after exposure to a liquid is compared to the change in area under the peaks of Fe, Ni, and Zn. Comparison of these parameters allows for analysis of variability between experiments.

**Figure 1:**
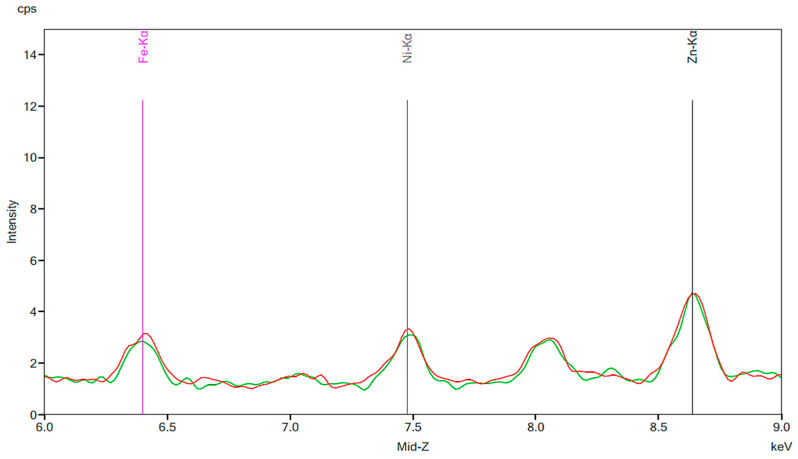
The results of the control group. The red peaks correspond to the baseline of the new artificial teeth prior to exposure to water. Markers are shown for iron, nickel and zinc. The green peaks represent the same artificial teeth following exposure to tap water for 24 h.

**Figure 2:**
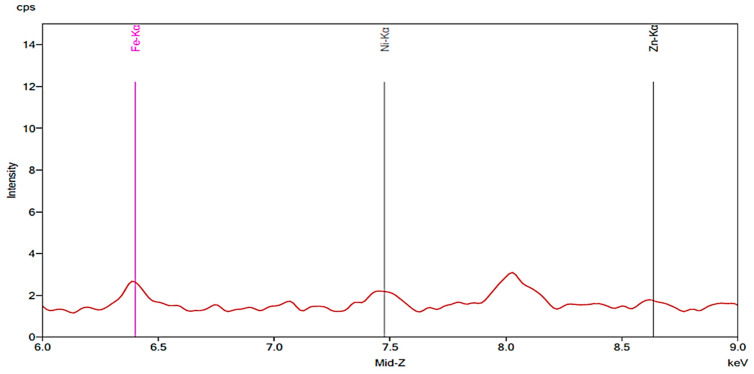
The figure is a portion of the spectrum for a sample of the coffee which was used for the experiments discussed in this paper. This portion is taken from energies ranging from 6.0 KeV to 9.0 KeV plotted against intensity on the y-axis in units of counts per second (cps).

**Figure 3:**
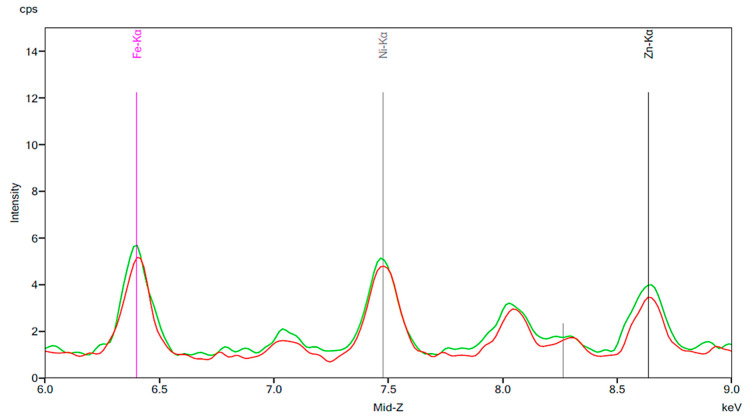
Spectrum displaying the results of an experiment in which new artificial teeth were exposed to coffee. The red peaks correspond to the new artificial tooth before being exposed to coffee. The green peaks represent the same artificial tooth following 24 h of exposure to coffee.

**Figure 4:**
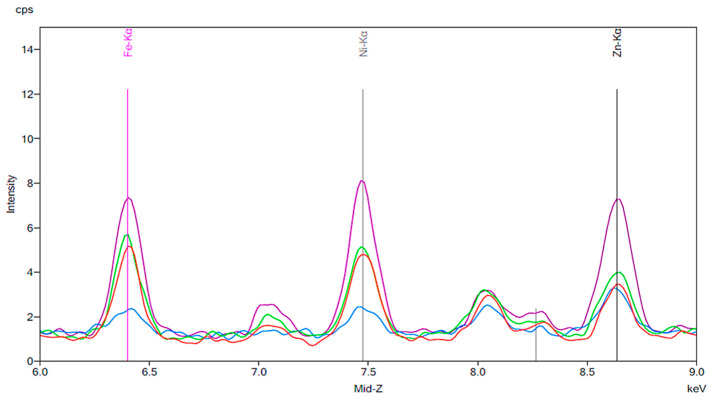
The same results as Figure 3 are indicated by the red and green peaks. The blue peaks on this figure represent a new artificial tooth that has been mechanically worn down. The purple peaks indicate the spectrum of an artificial resin tooth after wear following 24 h exposure to coffee.

Figure 1 illustrates the spectrum from the control group. The spectrum is notable for the presence of iron, nickel, and zinc. There is no significant change before and after 24 h exposure to tap water. Figure 2, Figure 3 and Figure 4 demonstrate the spectra from the experiments after coffee exposure for 24 h. Figure 2 shows the presence of iron, nickel, and zinc in a sample of the coffee used for these experiments. Figure 3 displays the spectrum peaks for artificial teeth before and after exposure to the coffee sample for 24 h. A small increase in the intensity of the peaks corresponding to the three metals was observed. The peaks shown in Figure 3 are displayed once again in Figure 4. Additionally, peaks for artificial teeth that were worn down are shown. A substantial increase in iron, nickel, and zinc was noted for the worn-down artificial teeth compared to the new teeth. In the case of the worn-down teeth, there is approximately a 3- to 4-fold change for the three metals. 

**Figure 5:**
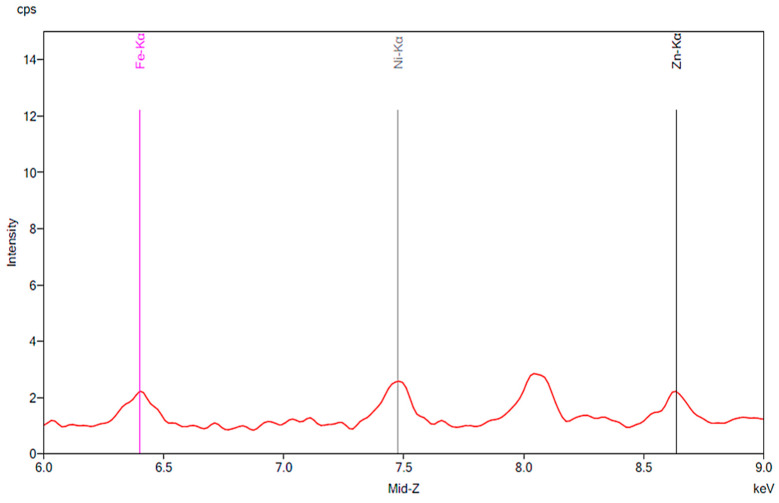
Baseline spectrum for a sample of cola. Similar units and scales are used for this figure as those shown in Figure 1.

**Figure 6:**
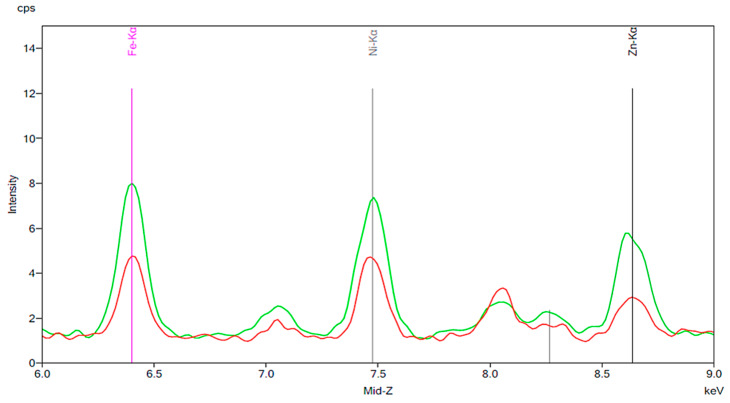
The results of an experiment in which new artificial resin teeth were exposed to cola for 24 h. The red peaks represent the new artificial tooth prior to exposure to cola. The green peaks indicate the same artificial tooth following exposure to cola for 24 h.

**Figure 7:**
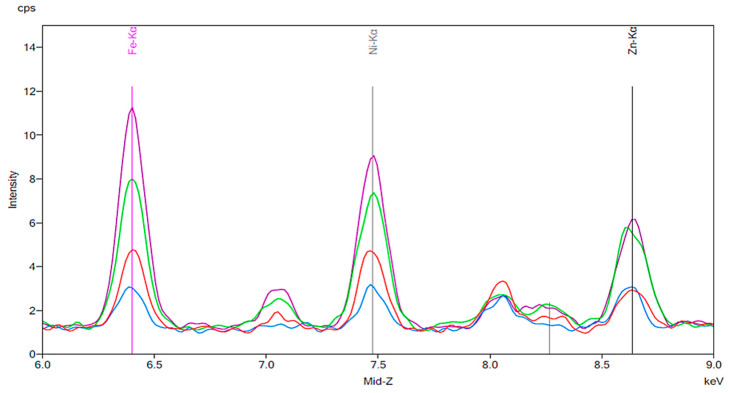
The same results as Figure 6 are indicated by the red and green peaks. For this experiment, the blue peaks represent a new artificial tooth after wear. The purple peaks correspond to the same artificial tooth following 24 h exposure to cola fluid.

Figure 5, Figure 6 and Figure 7 are similar to Figure 2, Figure 3 and Figure 4. A baseline for a sample of cola is shown, followed by the results of exposing new artificial teeth to the cola for 24 h. Finally, the peaks from Figure 6 are shown again on Figure 7 where they can be compared to the peaks corresponding to artificial teeth after wear and exposure to the cola liquid. A substantial increase in iron, nickel, and zinc is seen on Figure 6 compared to the peaks shown in Figure 3 for the new artificial teeth exposed to coffee. Here, there is approximately a 1.5 to 2-fold increase in the three metals following exposure to cola for 24 h. Similar to the results seen in Figure 4, the increase in the three metals is more substantial for the artificial resin teeth after wear than completely new resin teeth; Figure 7 shows a 4- to 5-fold increase in iron and nickel as opposed to the 1.5- to 2-fold increases seen in Figure 6.

**Figure 8:**
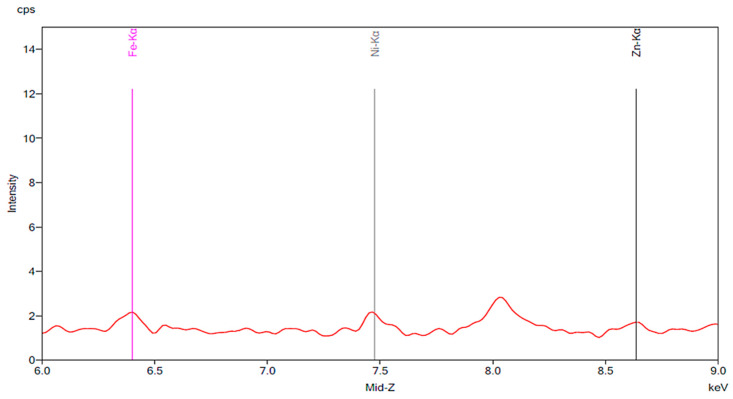
Baseline spectrum for a sample of black tea. The same parameters are used as those shown in Figure 2 and Figure 5.

**Figure 9:**
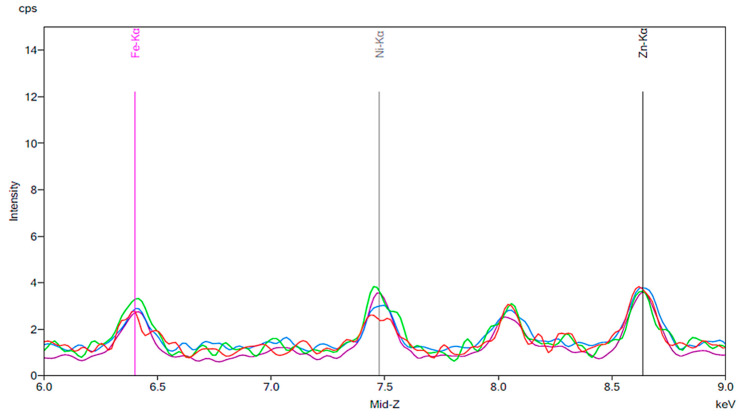
The results of an experiment in which new artificial teeth and artificial teeth after wear were exposed to tea. The red peaks and the green peaks correspond to the new artificial tooth with no wear before and after exposure to tea for 24 h, respectively. The blue and purple peaks represent the worn-down artificial tooth before and after 24 h of exposure to tea, respectively.

Figure 8 and Figure 9 are the spectra for the experiments performed using tea. While Figure 8 shows the presence of iron, nickel, and zinc in a sample of tea, Figure 9 shows only small increases in iron and nickel intensity following 24 h of exposure. This is the case for both new artificial teeth and teeth after wear.

**Figure 10:**
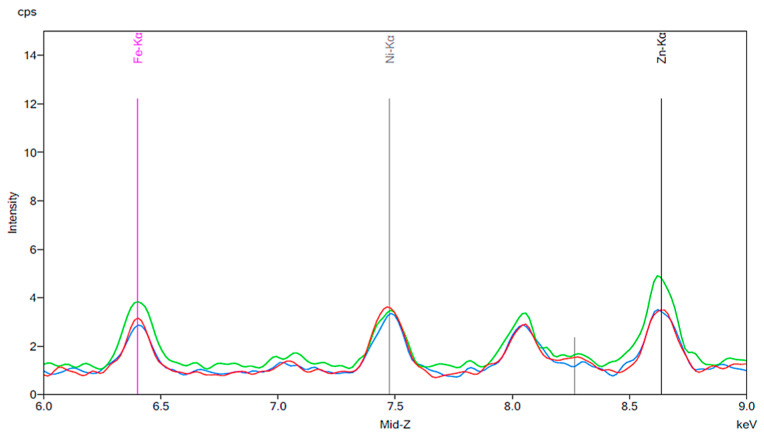
The results of an experiment in which artificial teeth were exposed to tea for 24 h. These teeth were then cleaned with a toothbrush for 30 s. The red peak represents the artificial tooth before exposure to tea. The green peak corresponds to the same tooth following 24 h exposure to tea. The blue peak represents the same tooth that had been exposed to tea following 30 s of brushing.

**Figure 11:**
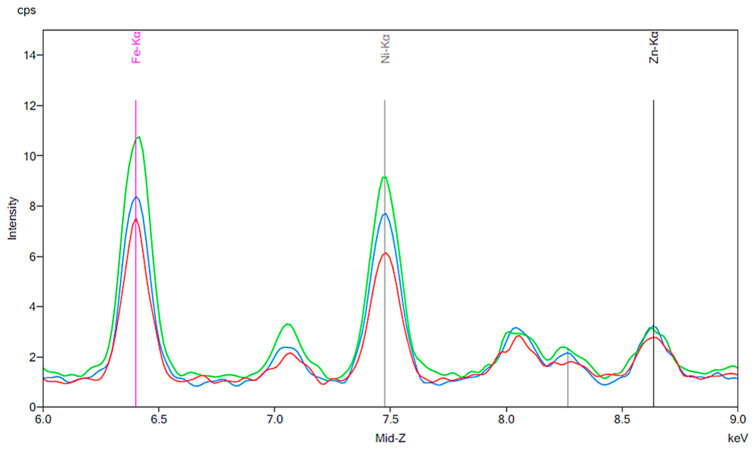
The results of the same tooth brushing experiment as described for Figure 10 used for teeth exposed to coffee. The red peaks indicate the new artificial tooth. The green peaks show the artificial tooth exposed to coffee for 24 h. The blue peaks represent the coffee exposed tooth following 30 s of brushing.

**Figure 12:**
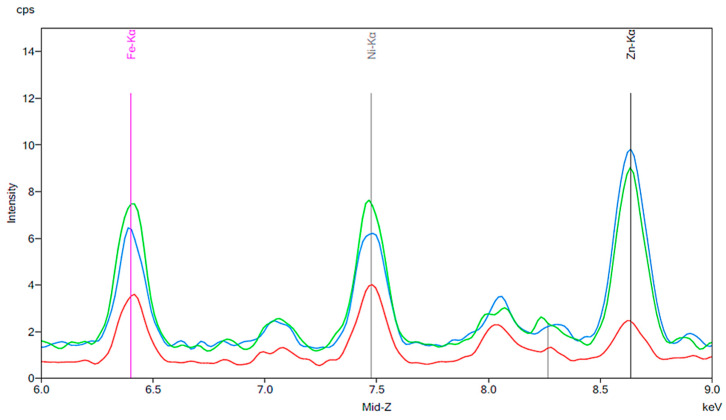
The results of the tooth brushing experiment for artificial teeth exposed to cola. The red peaks indicate new artificial teeth. The green peaks represent the artificial teeth after 24 h exposure to cola. The blue peaks correspond to the cola exposed tooth following 30 s of brushing.

**Figure 13:**
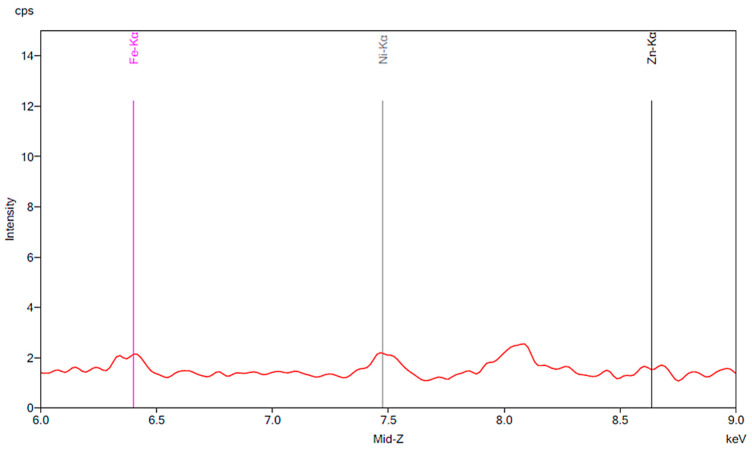
Baseline spectrum for a sample of chlorhexidine. The parameters are the same as those used for the other baseline spectra described in this paper.

**Figure 14:**
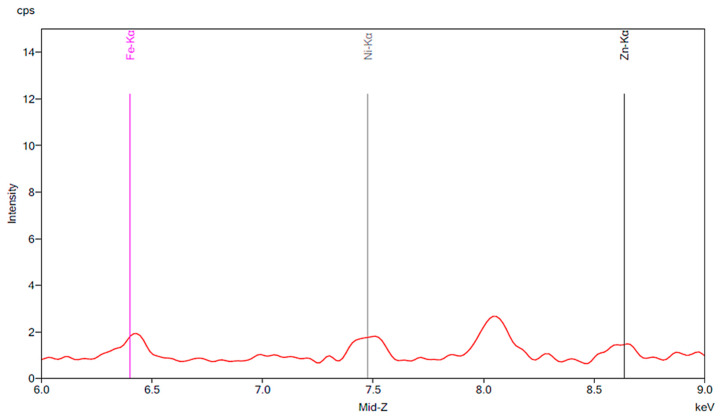
Baseline spectrum for a sample of red wine. The parameters used are the same as those described for the other baseline spectra previously discussed in this paper.

**Figure 15:**
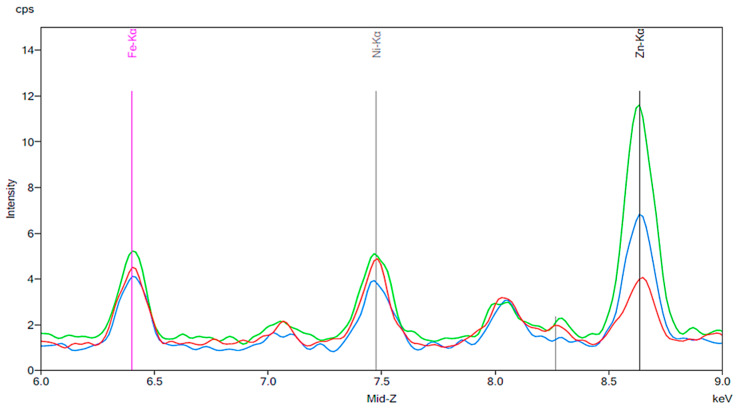
The results of the brushing experiment for artificial teeth exposed to chlorhexidine. The red peaks indicate new artificial teeth. The green peaks represent the artificial teeth following 24 h of exposure to chlorhexidine. The blue peaks correspond to the chlorhexidine exposed teeth following 30 s of brushing.

**Figure 16:**
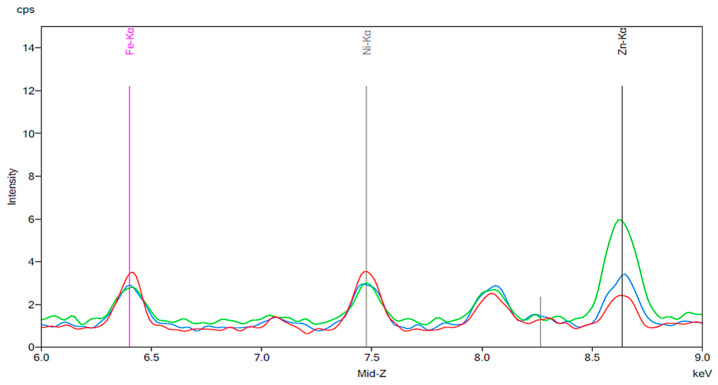
The results of the brushing experiment for artificial teeth exposed to red wine. The red peaks correspond to the new artificial teeth. The green peaks represent the artificial teeth following 24 h exposure to red wine. The blue peaks indicate the red wine exposed teeth following 30 s of brushing.

Figure 10, Figure 11, Figure 12, Figure 13, Figure 14, Figure 15 and Figure 16 show the results of the experiments in which artificial resin teeth were exposed to a beverage or other liquid for 24 h and then cleaned with a toothbrush. Figure 10 shows a slight increase in intensity for iron and zinc following 24 h of exposure to tea. While these increases were relatively small, it is notable that the resulting peaks decreased such that they overlapped with the peaks of the new artificial teeth prior to exposure to tea after the teeth were brushed for 30 s. 

Figure 11 and Figure 12 are the results of the same brushing experiment performed on denture teeth exposed to coffee and cola, respectively. As noted previously, cola and coffee exposure led to substantial increases in peak intensity for iron, nickel, and zinc. Figure 11 and Figure 12 show a reduction in intensity for iron and nickel following 30 s of brushing the exposed denture teeth. The brushing experiments were also performed on artificial teeth exposed to chlorhexidine and red wine. The baselines for chlorhexidine and red wine are shown in Figure 13 and Figure 14, respectively. The presence of iron, nickel, and zinc is notable in both spectra. The most substantial increase in intensity shown in Figure 15 corresponds to zinc. The intensity of this peak went from approximately 4 cps to 12 cps before and after exposure to chlorhexidine for 24 h. The peak intensity then decreased to about 7 cps following 30 s of brushing. Similar to Figure 15, the most significant change seen in Figure 16 is in the peak corresponding to zinc. The intensity of the peak increased by about 3-fold after the artificial teeth were exposed to red wine for 24 h. Once again, a decrease in the intensity is notable following 30 s of brushing.

## 4. Discussion

This is an important study, as many fixed dental prostheses are composed of nickel-titanium or nickel-chromium alloys [11]. Previous research has shown that, under certain conditions, the release of metal ions from these alloys can be amplified [9]. Chlorhexidine is frequently given to patients for use following periodontal or implant surgery in order to prevent biofilm growth and postoperative infections, however, research on the efficacy of chlorhexidine as a prophylactic agent is ongoing [12,13]. It is for this reason that we selected chlorhexidine as one of the liquids investigated in this study. Coffee, cola, tea, and red wine were each investigated as well, as they are all popular beverage choices for much of the general population despite the well documented oral and systemic health effects of consuming these beverages [14].

We chose to expose the artificial teeth to each liquid for 24 h in order to evaluate the effect of prolonged exposure on the surface of the teeth. This kind of prolonged exposure may take place in patients who consume one of these beverages before going to bed. If these patients do not clean or brush the artificial teeth afterwards, the residue of the liquid may then be left on the surface of the teeth overnight. Similarly, these patients may consume one of these beverages in the morning and then proceed for a long period of time without brushing or rinsing the teeth, leading to a greater period in which metal accumulation on the surfaces may occur.

The present study supported the conclusions of several similar studies, and it also shows some other relationships that have not been documented as much in literature. Figure 1 validates the efficacy of spectrometry for this study. It can be concluded that the spectrum of a given artificial tooth will show little to no change between different trials, even when exposed to tap water for 24 h. To further account for any variability that may have occurred between experimental trials, the changes in areas under the peaks of Fe, Ni, and Zn were compared to the change in area under the peak of Cu. These fold changes are shown in Table 1. We can therefore conclude that the results seen when artificial resin teeth were exposed to other liquids such as cola and coffee were due to these variable liquids rather than fluctuations in the machine or the procedure. The spectra shown in Figure 5 and Figure 6 support the conclusions of a study completed by Mikulewicz et al. which utilized an inductively coupled plasma - optical emission spectrometry (ICP-OES) spectrometric analysis. This is a similar method to the one utilized in our study in that it is an elemental analysis of certain materials, however ICP-OES spectrometry utilizes a plasma medium to dissolve and ionize materials. It is considered a complementary analysis to X-ray fluorescent spectrometry, so it is significant that the results from our study support the conclusions of their research. Their results suggest that cola increases the release of Ni and Fe ions from dental appliances. This is significant as nickel can be toxic in some patients [15,16]. 

Many studies have been performed to investigate the efficacy of chlorhexidine use. It can be used safely in patients with dental restorations in that the surface characteristics such as frictional resistance and roughness of the appliances is not significantly harmed by the chlorhexidine [13,17]. Our study confirms that chlorhexidine may increase the release of metal ions from dental appliances (Figure 13 and Figure 14). This supports the conclusions of a study performed by Danaei et al. that chlorhexidine use does correlate to higher ion release than other mouthwashes [18]. For this reason, it is important that physicians consider the degree of ion release and potential hypersensitivity in some patients when giving prescription mouth rinses [9,19]. 

In addition to our data on cola and chlorhexidine, the spectrometric analysis shown in this paper indicates that coffee, black tea, and red wine may each be associated with increased ion release from artificial resin teeth as well. The observation that these liquids may increase a patient’s exposure to metals such as iron, nickel, and zinc are significant due to the potential toxicity of these metals. The placement of dental appliances alone is associated with metal ion release into saliva. The number of ions released is typically well below the toxic limit under normal conditions, but complications may arise in patients with hypersensitivity, particularly to nickel [20,21]. Hypersensitivity to Ni is a type IV T-cell mediated delayed-onset hypersensitivity [22]. Adverse reactions to nickel are well documented in several case studies. Ehrnrooth and Kerosuo reported a 34-year-old female patient who presented with dermatitis of the face and neck following the placement of a maxillary expansion appliance. The patient’s symptoms disappeared a few days after the appliance was removed, and she was sent for a patch test for nickel hypersensitivity which came back positive [23]. Kolokitha and Chatzistavrou reported a more severe case in which a patient presented with eczema and urticaria of the face as well as intraoral red zones following surgical exposure and placement of orthodontic appliances. This patient later tested positive for Ni hypersensitivity [24]. 

After investigating the role of chlorhexidine and popular beverages in releasing metal ions into saliva, we performed experiments to observe the possible effects of abrasion and cleaning with a toothbrush. Many investigators have conducted studies on denture wear as a large population of patients may wear down their natural teeth or artificial resin teeth through parafunctional habits such as bruxism [10]. We decided to run our experiments on artificial teeth which were worn down using a dental handpiece in order to simulate conditions which are more realistic for a patient who has worn down their prosthetic teeth over time through masticatory function. We concluded, based on the results shown in Figure 4 and Figure 7, that worn-down artificial teeth were more susceptible to an increase in metal ions than new artificial teeth. While the mechanism behind this observation is not well understood, these results are significant, especially when considering patients who are prone to parafunctional habits and are hypersensitive to these metals. 

A significant portion of the older population is made up of dental patients with fixed or removable artificial teeth due to one or more missing teeth. The beneficial effects of proper cleaning, such as reduced formation of biofilm on the surfaces of artificial teeth, are well known, but many patients do not carry out proper cleaning on a daily basis [1,2]. Many of the beverages that were tested in our study, such as cola and coffee, are popular among the general population [25]. The results shown in Figure 10, Figure 11 and Figure 12, Figure 14, and Figure 16 show an increase in metal ion concentration on artificial teeth following exposure to popular beverages as well as chlorhexidine. It was observed that brushing the artificial teeth for just 30 s under tap water significantly reduced the amount of metal ions that had accumulated. This observation strongly emphasizes the importance of proper care for dental appliances, especially following exposure to coffee, cola, black tea, red wine, or chlorhexidine. 

When analyzing the results of this study, it is worth considering other variables that may have impacted the final outcome. One important consideration is the amount of metal generated during the manufacturing process of the artificial resin teeth. This could potentially vary for each individual tooth and may have impacted the results seen in the study. It is for this reason that a semi-quantitative analysis with a focus on changes in peak intensities for each metal was utilized. Even though the teeth may have differed from one another in initial concentrations of iron, nickel, and zinc, the spectra show increases in the peak intensities following exposure to some of the liquids, regardless of the initial peak intensities. The inability to precisely quantify the concentrations of each metal could be a shortcoming of this study, and future studies are likely needed to determine the exact extent to which metal accumulation on the artificial teeth surfaces is occurring using specific laboratory methods. 

## 5. Conclusions

This study showed a pronounced accumulation of metal ions such as iron, nickel, and zinc on the surface of artificial teeth following exposure to cola, coffee, black tea, chlorhexidine, and red wine. The increase in concentration of the metals in relation to peak intensity varied between the different liquids. Artificial teeth which were mechanically worn-down showed a noticeably larger increase in peak intensities than brand new artificial teeth. Cleaning an artificial resin tooth with a toothbrush for 30 s resulted in decreased peak intensities of the accumulated metal ions.

## Figures and Tables

**Table 1:** Average data collected over multiple trials for each experimental group. Mean fold changes in peak intensity were calculated using the spectrometer software which allows the operator to extract peak values for each element within a spectrum. Mean fold changes which compare the change in area under the peak corresponding to Fe, Ni, or Zn to the change in area under the peak for Cu are also shown. The spectra shown throughout this paper come from one of the trials for each experimental group.

Experimental Group	Tooth Surface	Element	Mean Fold Change in Peak Intensity	Mean Fold Change in (∆A under peak)/(∆A under Cu Peak)
		Fe	1.1 ± 0.13	1.0 ± 0.05
Control/Tap Water	Original	Ni	1.0 ± 0.05	1.1 ± 0.08
		Zn	1.0 ± 0.17	1.0 ± 0.05
		Fe	1.4 ± 0.38	1.1 ± 0.33
Coffee	Original	Ni	1.4 ± 0.40	1.0 ± 0.29
		Zn	1.2 ± 0.14	1.2 ± 0.12
		Fe	4.0 ± 0.13	3.7 ± 0.52
Coffee	Worn Down	Ni	4.0 ± 0.15	3.7 ± 0.47
		Zn	2.0 ± 0.14	1.9 ± 0.22
		Fe	2.0 ± 0.21	1.8 ± 0.12
Cola	Original	Ni	2.0 ± 0.23	1.8 ± 0.16
		Zn	2.2 ± 0.78	2.3 ± 0.57
		Fe	3.7 ± 0.53	3.9 ± 0.40
Cola	Worn Down	Ni	3.2 ± 0.17	3.4 ± 0.12
		Zn	2.8 ± 0.29	2.7 ± 0.35
		Fe	1.1 ± 0.21	1.1 ± 0.15
Tea	Original	Ni	1.2 ± 0.15	1.0 ± 0.40
		Zn	1.2 ± 0.33	1.2 ± 0.22
		Fe	1.0 ± 0.05	1.1 ± 0.21
Tea	Worn Down	Ni	1.1 ± 0.12	1.2 ± 0.13
		Zn	1.1 ± 0.14	1.1 ± 0.25

**Table 2:** Average data collected for each experimental group for the brushing experiments. Percentage reductions in peak intensity following brushing were calculated based on the extent to which the intensity returned to baseline (the quantity before exposure to the liquid) following 30 s of brushing. A value of N/A indicates that there was no substantial increase in peak intensity following exposure to the liquid, therefore a reduction in intensity following brushing is non-applicable.

Experimental Group	Element	Percentage Reduction in Peak Intensity After Brushing
	Fe	100 ± 2.2
Tea	Ni	N/A
	Zn	100 ± 2.5
	Fe	50 ± 4.8
Coffee	Ni	50 ± 4.0
	Zn	N/A
	Fe	25 ± 5.4
Cola	Ni	25 ± 4.7
	Zn	0 ± 4.4
	Fe	100 ± 5.6
Chlorhexidine	Ni	N/A
	Zn	50 ± 4.5
	Fe	N/A
Red Wine	Ni	N/A
	Zn	75 ± 5.8
